# The Prevalences of Salmonella Genomic Island 1 Variants in Human and Animal *Salmonella* Typhimurium DT104 Are Distinguishable Using a Bayesian Approach

**DOI:** 10.1371/journal.pone.0027220

**Published:** 2011-11-18

**Authors:** Alison E. Mather, Matthew J. Denwood, Daniel T. Haydon, Louise Matthews, Dominic J. Mellor, John E. Coia, Derek J. Brown, Stuart W. J. Reid

**Affiliations:** 1 Boyd Orr Centre for Population and Ecosystem Health, College of Medical, Veterinary and Life Sciences, University of Glasgow, Glasgow, United Kingdom; 2 Scottish Salmonella Shigella and Clostridium difficile Reference Laboratory, Stobhill Hospital, Glasgow, United Kingdom; University of Oxford, Viet Nam

## Abstract

Throughout the 1990 s, there was an epidemic of multidrug resistant *Salmonella* Typhimurium DT104 in both animals and humans in Scotland. The use of antimicrobials in agriculture is often cited as a major source of antimicrobial resistance in pathogenic bacteria of humans, suggesting that DT104 in animals and humans should demonstrate similar prevalences of resistance determinants. Until very recently, only the application of molecular methods would allow such a comparison and our understanding has been hindered by the fact that surveillance data are primarily phenotypic in nature. Here, using large scale surveillance datasets and a novel Bayesian approach, we infer and compare the prevalence of Salmonella Genomic Island 1 (SGI1), SGI1 variants, and resistance determinants independent of SGI1 in animal and human DT104 isolates from such phenotypic data. We demonstrate differences in the prevalences of SGI1, SGI1-B, SGI1-C, absence of SGI1, and tetracycline resistance determinants independent of SGI1 between these human and animal populations, a finding that challenges established tenets that DT104 in domestic animals and humans are from the same well-mixed microbial population.

## Introduction

Multidrug resistant *Salmonella* Typhimurium DT104 was first identified in the 1980 s [1]; following its identification it became one of the most commonly isolated serovars of Salmonella from cases of human and animal clinical infection over the following two decades in Europe and North America [2]. Its zoonotic potential and epidemiology are well established; cattle density and contact with cattle or other animals are identified risk factors for human infection with DT104 in the UK [3]–[5] and elsewhere [6]–[8]. The epidemic of DT104 that occurred in Scotland from the late 1980 s to the mid 2000 s provides an opportunity to chart the rise and fall of a zoonotic pathogen in both humans and animals. Furthermore, as a clonal bacterial infection that is frequently multiresistant to antimicrobials, and one that is reportable in both medical and veterinary domains, the identification and distribution of resistance phenotypes between species is also possible. This is important because the agricultural and veterinary use of antimicrobials is often cited as a major driver of the emergence and spread of multidrug resistant bacteria in humans [9]–[13]. A key issue in considering the relative contribution to antimicrobial resistance (AMR) in both animals and humans is the similarity, or otherwise, of the distribution of resistance phenotypes, and resistance determinants in the two populations. This question can only be addressed by the use of sympatric and contemporaneous surveillance data.

There are several resistance phenotypes of DT104 described in the literature. The most common resistance pattern involves resistance to ampicillin (A), chloramphenicol (C), spectinomycin (Sp), streptomycin (St), sulphonamides (Sx) and tetracycline (T), which are chromosomally encoded, the genes residing in a region termed Salmonella genomic island 1 (SGI1) [14]. Although phenotypic resistance to A, C, Sp, St, Sx, and T strongly suggest that SGI1 is present in a DT104 isolate [15], [16], it is possible that these resistances arose independently and the genes are located outwith SGI1. As a consequence, hitherto it has not been possible to determine the genotype of an isolate given its phenotype, as several different genes with distinct resistance mechanisms may be responsible for the same phenotype. This is unfortunate as most AMR data collected are phenotypic, particularly in clinical surveillance systems. Whilst high throughput genotyping does offer a solution to this problem, cost and availability currently limit the opportunities to exploit fully this technology. Here, using a method capable of inferring genotypes from phenotypic data [17], we assess the prevalence of SGI1 and its variants, as well as the prevalence of SGI1-independent resistance determinants, in sympatric and contemporaneous animal and human populations, thereby contrasting the epidemics of the organism in two species. In doing so, we examine established tenets that DT104 in domestic animals and humans are from the same well-mixed microbial population.

## Materials and Methods

### Data collection

The data used in this study were part of an ongoing passive surveillance effort for Salmonella. A total of 2,439 animal and 2,761 human *Salmonella* Typhimurium DT104 isolates were submitted to the Scottish Salmonella, Shigella and Clostridium difficile Reference Laboratory (SSSCDRL) from 1990 to 2004, covering the epidemic period of DT104. Identified human Salmonella isolates from diagnostic medical laboratories were sent to the SSSCDRL for confirmation and further testing, and animal isolates were submitted to the SSSCDRL from veterinary laboratories. The SSSCDRL is fully accredited with Clinical Pathology Accreditation (UK) Ltd. Only human isolates from domestic cases with no recent history of foreign travel were included in this study. Serotyping of the isolates was accomplished according to internationally standardised methods based on the Kauffman-White scheme [18], and phage typing was carried out according to standardised protocols [19], [20] at the SSSCDRL.

### Susceptibility testing

Antimicrobial susceptibility was assessed at the SSSCDRL for both animal and human isolates using a modified breakpoint method, involving solid agar plates containing a pre-determined concentration of antimicrobial. The breakpoint for ampicillin was 50 µg/mL; chloramphenicol, 20 µg/mL; spectinomycin, 100 µg/mL; streptomycin, 20 µg/mL; sulphamethoxazole, 100 µg/mL; tetracycline, 10 µg/mL. Suspensions of Salmonella were inoculated onto the surface of the plates; bacterial growth, following incubation, indicated resistance to that antimicrobial at that concentration [21]. Categories of resistance to each antimicrobial were non-resistant or resistant, with a resistance profile defined as a unique combination of resistances.

### Assessing linkages between antimicrobial resistances

A full description of the statistical techniques and program code is given in Denwood et al. [17]. Briefly, Bayesian Markov chain Monte Carlo (MCMC) methods were used to extract information about the hidden variable state (resistance genotype), from the observed variable state (resistance phenotype), using a hierarchical mixture model. The goal was to estimate the prevalence of SGI1 and each of the SGI1 variants of DT104 previously identified in the literature (Table 1) [22]–[32], and the prevalence of resistance determinants outside of SGI1 in both the animal and human datasets. Variants found in other Salmonella serovars, or other bacteria, were excluded, and the presence of resistance genes to antimicrobials other than the six antimicrobials in the full SGI1 (A, C, Sp, St, Sx, T) were not considered.

**Table 1:** SGI1 and SGI1 variants found in *Salmonella* Typhimurium DT104 with their resistance phenotypes to the six antimicrobials in the full SGI1 [ampicillin (A), chloramphenicol (C), spectinomycin (Sp), streptomycin (St), sulphonamides (Sx) and tetracycline (T)] and resistance genes as reported in the literature.

Variant name	Variant number	Phenotype	Resistance genes	Reference
Absence of SGI1	1	–	–	–
SGI1	2	ACSpStSxT	*bla*_PSE-1_, *sulI*, *aadA2*, *floR*, *tetR*, *tetG*	[23]
SGI1-B	3	ASx	*bla*_PSE-1_, *sulI*	[22], [25]–[27], [29], [30]
SGI1-C	4	SpStSx	*aadA2*, *sulI*	[22], [24], [26], [27], [29], [30], [32]
SGI1-E	5	ASpStSxT	*bla*_PSE-1_, *sulI*, *aadA2*, *floR* (interrupted), *tetG*	[22]
SGI1-M	6	ACSxT	*bla*_PSE-1_, *floR*, *tetR*, *tetG*, *sulI*, *aadB* *	[31]
SGI1-T	7	CSpStT	*floR*, *tetR*, *tetG*, *aadA2*	[28]

*conferring resistance to kanamycin, gentamicin, and tobramycin; this information not used.

For each antimicrobial, *a* 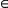 {1,2,3,4,5,6} (where 1 = Ampicillin, 2 = Chloramphenicol, 3 = Spectinomycin, 4 = Streptomycin, 5 = Sulphonamides and 6 = Tetracycline), tested for each isolate, *i*, the probability of observing a resistance to antimicrobial *a* in isolate *i*, *P*(*R_ai_*), is given by:
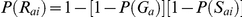
where *S_a_* refers to observation of resistance to *a* when it is encoded by SGI1, and *G_a_* refers to the observation of resistance to *a* when it is encoded elsewhere on the genome. The latter event represents the combined probability of one or more such genes existing and the probability of phenotypic expression of these genes given that they exist.

The probability of observing a resistance encoded by SGI1, *P*(*S_ai_*), for each antimicrobial is now conditional on the type of SGI1 variant, *V* 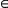 {1,2,3,4,5,6,7} (see Table 1), as well as depending on the probability of expression for that antimicrobial, *E_a_*. For each isolate, *i*,
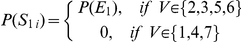


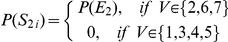


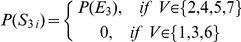


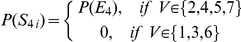


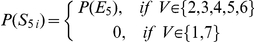


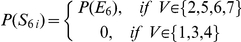
The probabilities *P*(*G_a_*) and *P*(*E_a_*) are estimated using minimally informative Beta priors and the multinomial probability vector *P*(*V = v*) for *v* = 1,…,7 estimated using a minimally informative Dirichlet prior [17]. Although resistance to Sp and St is mediated by the same gene (aadA2) in SGI1, Sp and St resistances are included in the model individually. This is partly due to the different phenotypic breakpoints used for the two antimicrobials; by including both Sp and St separately, we allow for the possibility of a profile that has phenotypic resistance to one but not the other. These resistances could be either due to a SGI1 variant (where the gene is present, but its phenotypic expression is not sufficiently high enough for one to be detected), or to be due to genes outside of SGI1. The pseudo-parameters P(S_3i_) and P(S_4i_) are presented separately above, but the two are inseparably linked due to being dependent on the same gene, i.e., if P(S_3i_) = 0, then P(S_4i_) = 0. However, if the two are not equal to zero then each maps directly onto the corresponding probability of phenotypic expression P(E_3_) and P(E_4_), which represents the possibility of a difference in phenotypically detecting the presence of the gene, given that the breakpoints are different for the two antimicrobials.

The model allowed each observed phenotypic profile to occur as a result of: i) one of seven possible variants of SGI1 that have been previously reported in the literature (the full SGI1, five variants, and no SGI1); ii) as a result of individual resistance elements, not related to SGI1, to one or more of the six antimicrobials of interest; iii) as a combination of the two. Each resistance determinant outside of SGI1 was assumed to arise independently of the presence or absence of other phenotypic resistances. Minimally informative priors were used for the prevalences of the various SGI1 variants and the prevalences of resistance determinants outside of SGI1, thus ensuring the prevalences were determined predominantly by the data. The probabilities of phenotypic expression for resistance determinants within SGI1, given that the determinant was present, were assumed to be independent, and unrelated to the probabilities of phenotypic expression for resistance arising from a gene not linked to SGI1. The model was implemented by MCMC simulation, using seven chains with over-dispersed initial values. Convergence was determined by a Gelman Rubin statistic of less than 1.05 for each parameter, in addition to visual inspection of the trace plots for each parameter. Model goodness-of-fit was assessed using posterior predictive p-values based on the number of unique profiles, as described and discussed extensively by Denwood et al. [17]. Briefly, for the animal and human models separately, posterior predictive p-values were calculated using the output of the MCMC model as follows: The combination of parameter values from a single iteration were used to obtain 1,000 bootstrapped datasets equal in size to the observed sample size (2,761 for the human data, 2,439 for the animal data). The distribution of the number of unique resistance profiles per bootstrapped dataset was then compared to the observed number of unique profiles. Monte Carlo integration was used to obtain a p-value, reflecting the probability that a dataset as, or more extreme than, the observed dataset would be obtained using the parameter values at that iteration. This process was repeated for the combination of parameter values at a total of 1,000 iterations, and the resultant 1,000 p-values along with an ‘overall’ p-value were generated for each model.

## Results

**Figure 1:**
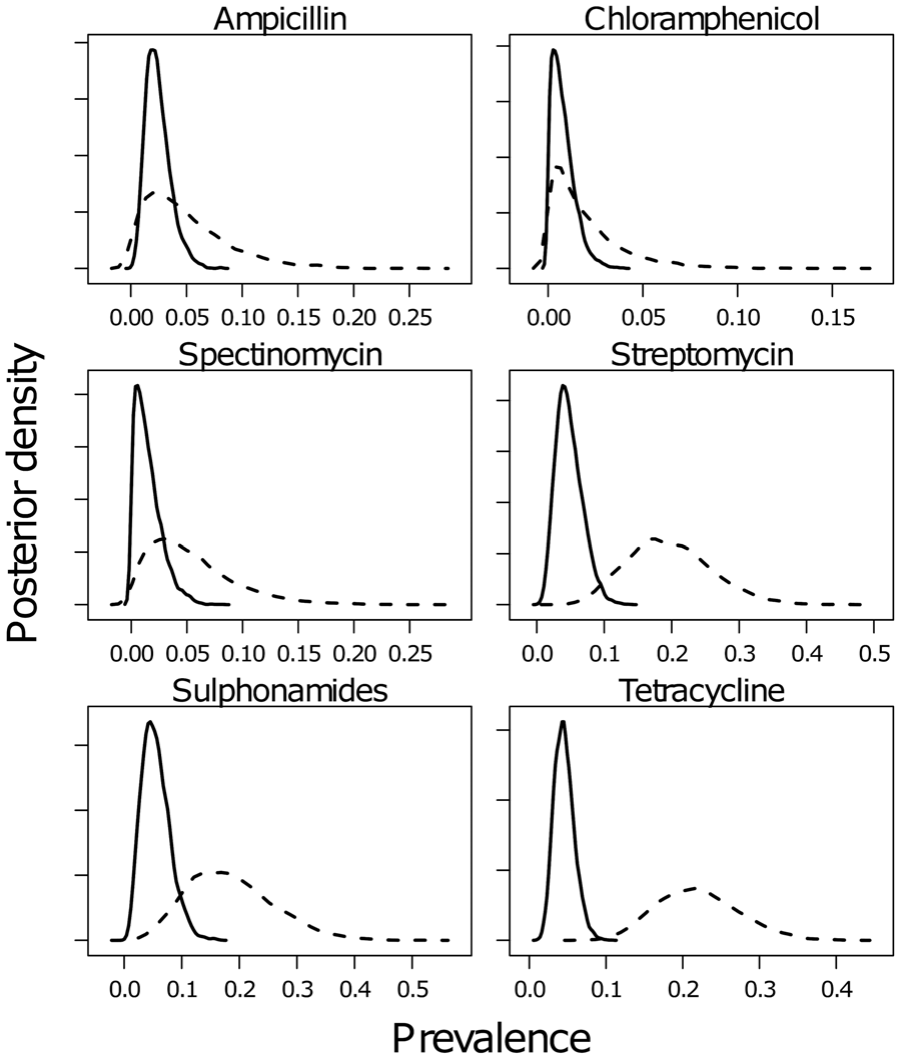
Predicted prevalences of resistance to individual antimicrobials. Posterior densities of the predicted prevalences of phenotypically expressed resistance determinants independent of SGI1 in Scottish *S.* Typhimurium DT104 data, 1990–2004, from animals (dashed line) and humans (solid line), to each of the six examined antimicrobials.

**Figure 2:**
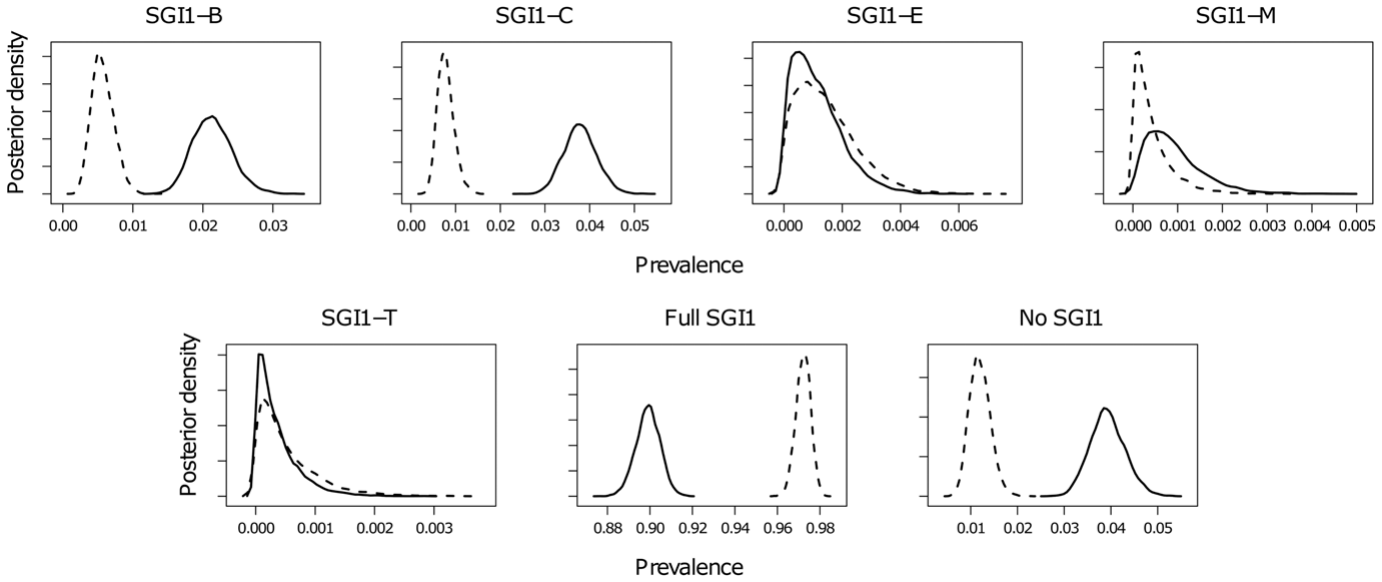
Predicted prevalences of SGI1 variants. Posterior densities of the predicted prevalences of SGI1, SGI1 variants, and absence of SGI1 in Scottish *S.* Typhimurium DT104 data, 1990–2004, from animals (dashed line) and humans (solid line).

The median and 95% credible intervals (CI) for each parameter estimated for the human and animal data are shown in Table 2. The predicted prevalences of the resistance determinants independent of SGI1 for both animal and human isolates are compared in Figure 1, and the predicted prevalences of SGI1 variants and the absence of SGI1 for both animal and human isolates are compared in Figure 2. The results of the analysis revealed differences in the estimated prevalences for certain resistance determinants between bacteria derived from animals and humans, namely, for three of the six SGI1 variants, and also the prevalence of isolates without SGI1. The median prevalence of the full SGI1 in human isolates was 0.90, whereas in the animal isolates, the median was higher at 0.97, with the 95% credible intervals non-overlapping. Conversely, in human isolates, the estimated prevalences of SGI1-B and SGI1-C were higher than in animal isolates, where for both variants the prevalences were estimated to be very low (95% CIs non-overlapping). SGI1-E, SGI1-M, and SGI1-T were both estimated to be present at extremely low prevalence or absent from both the human and animal isolate populations studied. In addition, the prevalence of tetracycline resistance associated with a gene outside the SGI1 was estimated to be higher in animal than in human isolates (95% CIs non-overlapping).

**Table 2:** The median and 95% credible interval (CI) estimates for each parameter estimated from the analysis of the 2,761 human and 2,439 animal *Salmonella* Typhimurium DT104 resistance profiles, for the six antimicrobials ampicillin (A), chloramphenicol (C), spectinomycin (Sp), streptomycin (St), sulphonamides (Sx), and tetracycline (T).

	Human	Animal
Parameter	Median (95% CI)	Median (95% CI)
Independent A	0.02 (0.01–0.05)	0.04 (0.00–0.14)
Independent C	0.01 (0.00–0.02)	0.01 (0.00–0.07)
Independent Sp	0.01 (0.00–0.05)	0.05 (0.00–0.15)
Independent St	0.05 (0.02–0.09)	0.19 (0.09–0.33)
Independent Sx	0.05 (0.02–0.11)	0.18 (0.06–0.35)
Independent T	0.04 (0.02–0.07)*	0.22 (0.13–0.33)*
No SGI1 variant	0.04 (0.03–0.05)*	0.01 (0.01–0.02)*
Full SGI1 variant	0.90 (0.89–0.91)*	0.97 (0.97–0.98)*
SGI1-B variant	0.02 (0.02–0.03)*	0.01 (0.00–0.01)*
SGI1-C variant	0.04 (0.03–0.05)*	0.01 (0.00–0.01)*
SGI1-E variant	0.00 (0.00–0.00)	0.00 (0.00–0.00)
SGI1-M variant	0.00 (0.00–0.00)	0.00 (0.00–0.00)
SGI1-T variant	0.00 (0.00–0.00)	0.00 (0.00–0.00)

*95% credible intervals non-overlapping between animal and human isolates.

‘Independent’ probabilities refer to the combined probability of a resistance determinant occurring outside of SGI1 and that being expressed in the phenotype. ‘Variant’ probabilities refer to the prevalence of each SGI1 variant.

The posterior predictive p-values for both the animal and human models were not statistically significant (p = 0.16 and p = 0.53, respectively), indicating that the models adequately describe the number of unique resistance phenotypes observed.

## Discussion

We demonstrate that during the epidemic of DT104 in Scotland, there were differences between isolates from animals and humans in the estimated prevalences of the three most common variants of SGI1 and of the absence of SGI1, as well as differences in the prevalence of tetracycline resistance determinants occurring independently of SGI1. The prevalence of the most common variant, the full SGI1, was estimated to be higher in animals than in humans, and was the most commonly predicted genotype in both populations. Although some may consider the estimated prevalences of SGI1 to be qualitatively similar, the fact that highly significant differences have been demonstrated between the populations is a strong indication that the two populations are not as well-mixed as has been suggested previously [10], [13]. The median prevalence of isolates not containing any SGI1 variant was 4% in humans, whereas in the animal isolates it was lower at 1%, with the 95% credible intervals non-overlapping. There were also differences in the prevalences of SGI1-B and SGI1-C between animal and human isolates, with the prevalences of both being higher in human isolates and there were differences in the prevalence of non-SGI1-related resistance determinants between the two populations. For example, the prevalence of tetracycline resistance due to a gene independent of SGI1 was higher in animal isolates than in human isolates. Established tenets, that the domestic animal population is a major source of the resistance in DT104 found in humans and that the microbial communities are well mixed, are widespread in the literature [10], [13], [33]–[35]. Were this the case we might expect similar prevalences of the various SGI1 variants and SGI1-independent resistance determinants in both animal and human isolates, but this was clearly not what we observed. Without making any claim regarding transmission, in either direction, of resistance determinants between the two populations, and acknowledging the existence of additional sources of resistance such as imported food, our observation does point to independent or differing processes occurring within each population during the epidemics with respect to evolution and dissemination.

The data we have used are the most suitable of any available for such a comparison. The isolates were collected from animals and humans from the same geographical area over the same time period, which covered the majority of the DT104 epidemic. They were also subject to the same microbiological and susceptibility testing techniques in the same laboratory; use of a single laboratory should minimise any bias relating to observed AMR differences between animal and human isolates, conditional on the method used. We infer genotype from the phenotype utilising the prior knowledge of the genetics of resistance in DT104, which has been very well characterised [14], [23], [34], [36]. Our approach represents a methodological advance in the analysis of phenotypic AMR data, allowing the prevalence of genetic elements to be inferred at the resolution of the population, rather than predicting the genotype of individual isolates. The ability to infer genotypic information from phenotypic data at this level will provide a greater insight into differences in the evolution and spread of resistance within and between populations.

In the context of DT104, it has been particularly interesting to have conducted this analysis on data spanning the entirety of the epidemic curve. However, a number of caveats remain. The nature of isolate collection and reporting present potential biases; because the isolates were passively collected and are likely to be predominantly diagnostic isolates from clinically ill individuals, it is possible that these results may not represent the characteristics of DT104 which are present in the general (healthy) population, although a recent study of *Salmonella* Typhimurium suggests that this may not be the case [37]. The differing prevalences of the various resistance determinants in animal and human DT104 isolates may be due to different antimicrobial usage patterns; it is possible that animals, having a higher prevalence of the full SGI1, are more likely than humans to have received antimicrobial treatment prior to a sample being submitted. However, there are no antimicrobial use data available to test a hypothesis regarding antimicrobial use and resistance. It is also possible that the disparity in results between the two populations could be due to a systematic difference in reporting of isolates in animals and humans, or to epidemiological differences in characteristics such as duration of infection or shedding patterns that may be affected by SGI1.

Nevertheless, these caveats are intrinsic to surveillance data of a phenotypic nature and, despite this, it is data of this kind that are used to support the alternative views and form the basis for policy [38], [39]. Molecular genotypic data would provide additional information on the comparison of the different genotypes in these populations and could be considered to be the gold standard. However, given the very low prevalence of the rarer phenotypes and inferred genotypes we describe here, the number of isolates that would be required to validate our approach would be unfeasibly large. In more general terms, our approach is advantageous because large-scale genotyping is, and has been, uncommon in surveillance programmes due to financial and logistical constraints, and is unlikely to be applied retrospectively. In addition, the methods we describe would be particularly relevant for the analysis of historic data where isolates are no longer available or viable.

In conclusion, we demonstrate differences between resistant *Salmonella* Typhimurium DT104 in human and animal populations during the epidemics that occurred in Scotland. These findings suggest that although these populations share a common ecological niche, the epidemiologies of the organism, the resistance determinants, or both, are distinguishable. Our approach has allowed previously unavailable data on genotype to be extracted from phenotypic data; based on this combination of phenotypic and genotypic data, our findings reinforce the need to understand the biology underlying the epidemics and the relative importance of each microbiological population in the different host species before implementing policy on antimicrobial usage.
